# Increased expression of fragmented tRNA promoted neuronal necrosis

**DOI:** 10.1038/s41419-021-04108-6

**Published:** 2021-08-30

**Authors:** Yanyan Cao, Kai Liu, Ying Xiong, Chunyue Zhao, Lei Liu

**Affiliations:** 1 Department of Biochemistry and Molecular Biology School of Basic Medicine, Capital Medical University, Youanmen, Beijing, China; 2 Department of Neurology, First Affiliated Hospital, and College of Clinical Medicine of Henan University of Science and Technology, Luoyang, China; 3 College of Life Sciences, Wuhan University, Wuhan, China; 4 Beijing Advanced Innovation Center for Big Data-Based Precision Medicine, Beihang University, Beijing, China

**Keywords:** Cell death, Cell death in the nervous system

## Abstract

Neuronal necrosis induced by excessive glutamate release is well known to contribute morbidity and mortality in ischemic stroke. Over the past decades, strategies on targeting glutamate receptor did not achieve desirable clinical outcomes. Finding the downstream mechanism of the glutamate receptor activation may provide new targets to suppress the cell death. Previously, our study demonstrated that the increase of H3K4 trimethylation (H3K4me3) played a key detrimental role on neuronal necrosis; however, the mechanism of this histone modification is unclear. Through a genome-wide small RNA sequencing, we identified several tRNA-derived fragments (tRFs) and piwi-interacting RNA (piRNAs) species were enriched in glutamate-induced neuronal necrosis in rat primary neuron cultures, and this enrichment was dependent on the H3K4me3 increase. Strikingly, when we transfected several synthesized tRFs and piRNA species into neurons, the tRFs but not the piRNAs induced neuron swelling and death. The cell death morphology recapitulated neuronal necrosis induced by glutamate. For the cytotoxic effect of tRFs, our data suggested that protein synthesis was inhibited likely through induction of ribosomal stalling. By proteomic analysis of tRFs effect, the most affected pathway was enriched in the mitochondrial metabolism. Consistently, mitochondrial fragmentation was increased in neuronal necrosis, and suppression of mitochondrial fission by genetic manipulation or drug rescued neuronal necrosis. Using our previously established *Drosophila* model of neuronal necrosis, we found that inhibition of small RNA transcription, blocking RNA transport from nucleus to cytosol, or knocking down *Ago1/2* to suppress the RNA interference effect, all rescued the fly death, suggesting transcription and processing of small RNAs contribute to neuronal necrosis. Together, these results indicate that the abnormal transcription of tRFs may play a key role downstream of the H3K4me3 increase. This provides a potential new strategy to suppress neuronal necrosis.

## Introduction

Excitotoxicity is induced by the excessive synaptic release of glutamate, which binds with various glutamate receptors, especially the N-methyl-D-aspartate (NMDA), to induce calcium overload and subsequent neuronal cell death. To suppress the excitotoxicity, many studies in the past had been focused on targeting the NMDA receptor, its plasma membrane translocation, its downstream calcium overload-induced calpain activity, or scavenger of oxidants; however, all these strategies did not gain desirable clinical efficacies [1–4]. Finding new solutions to prevent neuronal necrosis is still an urgent need in the field.

Previously, we established a genetic model of neuronal necrosis in *Drosophila* [5]. By genetic screens, we found that the increased H3K4 trimethylation (H3K4me3) by the Trithorax/Mixed lineage leukemia 1 (Trx/MLL1) contributed to neuronal necrosis in flies and mammals [5]. In mammals, six methytransferases including MLL1-4 and SET1A/1B are existed [6]. WDR5 is an essential component for these complexes [7]. We had previously designed a peptide (the experimental peptide, or Exp pep) to interfere the assembly of the WDR5 with MLL1, which could reduce the H3K4me3 mark and rescue the neuronal necrosis induced by glutamate. As a control, a double-mutant peptide (DM pep) that was unable to interfere with the WDR5/MLL1 complex, showed no rescue effect on neuronal necrosis [5]. Considering the H3K4me3 increase is a common marker of active transcription [8] and the inhibition of H3K4 methylation could rescue neuronal necrosis [5], we hypothesize that active transcription may produce some abnormal small RNAs which are cytotoxic. In fact, many non-coding RNAs (ncRNAs) are known to affect diverse physiological and pathological events, including transcription, post-transcription modulation, and formation of stress granules [9–11].

In the past decades, distinct ncRNAs have been identified to involve in the pathology of neurological disorders [12]. tRNA-derived fragments (tRFs, 12-50 nucleotides) are emerging as a new class of ncRNAs. The tRFs species are generally classified into five subtypes: tRF-5, tRF-3, tRF-1, tRF-2, and tRNA halves (tiRNA), based on its length, cleavage pattern, and sequence matched on the mature tRNA [13]. The tRF-5 and tRF-3 are derived from 5′ and 3′ ends of the mature tRNAs; whereas tRF-1 are derived from the 3′ ends of the pre-tRNA transcripts. The tRF-2 contains only the anticodon stem and loop tRNA [13]. The tiRNA are generated by specific cleavage in the anticodon loops of the mature tRNA [14, 15]. For the tRF function in neurodegeneration, one study showed that the accumulation of a tiRNA^tyr^ (46 nucleotides) played a key role in the degeneration of motor neurons. Strikingly, transfection of a synthetic tiRNA^tyr^ could induce apoptosis in the cultured neurons, suggesting this tiRNA^tyr^ species might be cytotoxic [16]. In addition, previous studies also reported that long noncoding RNA played a key role in cell death [17, 18]. Here, we explore the enrichment of small ncRNAs in neuronal necrosis.

## Materials and methods

### Data deposition

All the raw data has been deposited on Baidu Dbank (link: https://pan.baidu.com/s/1vInNoxTRlB8DrONxyDdTEg, Code: data).

### Fly maintenance and stocks

Flies were raised on standard sucrose/cornmeal medium at constant 18 °C or 25 °C with a 12 h light and dark cycle. The *UAS-GluR1*^*Lc.14*^ (on 2nd chromosome) was generated in our lab. The following lines were purchased from the Bloomington *Drosophila* Stock Center (Bloomington, IN, USA), including *Appl-Gla4* (on X chromosome, stock #32040), *tubulin-Gal80*^*ts*^ (on 2nd chromosome, stock #7019), *UAS-GFP* (on 3rd chromosome, stock #5430), *UAS-mitoGFP* (on 3rd chromosome, stock #8443), *Brf*^*c07161*^ (on 2nd chromosome, stock #17804), *dPolr3c*^*f05999*^ (on 3rd chromosome, stock #18929), *trx*^*KG08639*^ (on 3rd chromosome, stock #14748) and *Drp1*^*1*^(on 3rd chromosome, stock #24885). *Ran*^*G19V,Q69L*^ (on 2nd chromosome) was purchased from VDRC (Vienna Drosophila Resource Center, Austria). The RNAi (TRiP) lines were ordered from the Tsinghua *Drosophila* stock center (Beijing, China) including RNAi background control lines (TB00073 on 2nd chromosome and TB00072 on 3rd chromosome, these lines contain insertion of the transgenic vector into the defined location on chromosome 2 or 3, same insertion sites as the other RNAi transgenic lines), *Ago1RNAi* (on 2nd chromosome), *Ago2RNAi* (on 2nd chromosome) and *RanRNAi* (on 3rd chromosome). For the tests of the *AG* and *AGG* adult flies, flies with 1−3 days old were collected. Then, the flies were incubated at 30 °C for 24 h and then raised at 18 °C. After another 24 h, with lightly tapping the fly tube the movable flies were counted as viable. For the climbing assay, flies with 1−3 day old were collected and incubated at 30 °C for 12 h and then raised at 18 °C for 48 h. The ratio of flies reached on the top of a 10 cm tube within 30 s was calculated.

### Primary neuron culture

For the preparation of cortical neurons from embryonic mice (E18), cortical brain tissues were excised and were placed in an ice-cold DMEM (Dulbecco’s modified Eagle’s medium) (Gibco, USA), their meninges were removed and transferred into a 5 ml 0.25% trypsin dissociation medium. The tissue was slightly cut into small pieces and incubated for 10−20 min at 37 °C with constant shaking. Digestion was terminated in the DMEM with 10% fetal bovine serum, and triturated the mixture with a 10 ml pipette. The undigested tissue was allowed to sink in the bottom of the tube and the supernatant was collected into a sterile screw-capped tube and centrifuged at 1000 rpm for 5 min at room temperature. Discard the supernatant and resuspend the cell pellet in DMEM containing 10% fetal bovine serum (Gibco, USA) and 0.25% Penicillin-Streptomycin (Invitrogen, USA). The cells were filtered by a 40 μm Cell Strainer (BD Falcon, USA). The cells were then plated onto the poly-D-lysine-coated culture dishes and were maintained in the neurobasal medium with B27 (Invitrogen, USA), glutamax (Invitrogen, USA), and Penicillin-Streptomycin (Invitrogen, USA). After 48 h culture, 10 μM cytarabine was added in medium for 24 h to inhibit glial cell proliferation. The neuron cultures at days 7−10 were used for the glutamate treatment; while the transfections of ncRNAs were started at day 4 of the neuron culture.

### ATP and LDH release assays

Cellular ATP level was determined using a commercial kit (CellTiter-Glo^®^Luminescent Cell Viability Assay, Promega, USA) according to the manufacturer’s instruction. For the LDH release assay, the cell medium was collected first, then, the attached cells were lysed and collected. The LDH level was determined by a commercial kit (LDH Cytotoxicity Assay Kit, Beyotime, China). The cell death index was calculated as the following: LDH_medium_/(LDH_medium_ + LDH_cell_). Trial *n* = 2−4.

### tRFs sequencing

Total RNA was extracted by Trizol reagent (Invitrogen, USA) according to the manufacturer’s standard protocol. The purity and concentration of the RNA samples were determined with the NanoDrop ND-1000 (Thermo Scientific, USA). The rtStar™ tRF & tiRNA pretreatment kit (Arraystar, USA) was used to remove tRNAs with diverse modifications such as pseudouridine, 4-thiouridine, 1-methyladenosine, and queuosine. These modifications may interfere with the construction of the cDNA library. Total RNA of each sample was sequentially ligated to 3′ and 5′ small RNA adapters. Then, the cDNAs were obtained and amplified using the Illumina proprietary RT primers and amplification primers. Subsequently, ~134−160 bp PCR amplified fragments were extracted and purified from the PAGE gel. Finally, the completed libraries were quantified by an Agilent 2100 Bioanalyzer. The libraries were denatured and diluted to a loading volume of 1.3 ml with a concentration of 1.8 pM. Diluted libraries were loaded onto reagent cartridge and forwarded for sequencing on an Illumina NextSeq 500 system using the NextSeq 500/550 V2 kit (#FC-404- 2005, Illumina), according to the manufacturer’s instructions. Raw sequencing data generated from the Illumina NextSeq 500 which passes the Illumina chastity filter were used to the following analysis. Three independent samples were collected for each treatment. Data analysis workflow was listed as the following:

Trimmed reads (trimmed 5′, 3′-adaptor bases) were aligned to allow one mismatch to the mature tRNA sequences. Then reads that did not map were aligned to allow one mismatch to the precursor tRNA sequences by the bowtie software [19]. The remaining reads were aligned to allow one mismatch to the miRNA reference sequences with the miRDeep2 software [20]. Trimmed reads (with 5′, 3′-adaptor bases removed) were aligned to the piRBase (a manually curated resource of piRNAs) using the NovoAlign software. The alignment statistical analysis was applied to retain the valid sequences for the piRNA expression profiling and differential expression analysis. Based on alignment statistical analysis (mapping ratio, read length, fragment sequence bias), we determine whether the results could be used for the subsequent data analysis. The expression profiling and differentially expressed tRFs were screened based on the count value with R package edgeR [21]. The bubble chart is performed in R or perl environment for statistical computing and graphics of the expressed tRFs [21]. The software and database used in this study are listed:

(1) Softwares:

Perl: v5.16.3

Python: 2.7.5

R: 3.5.1

FastQC: v0.11.7

Cutadapt: 1.17

Bowtie: 1.2.2

NovoAlign: v2.07.11

miRDeep2: 2.0.0.8

(2) Databases:

GEO: http://www.ncbi.nlm.nih.gov/geo/

GtRNAdb: http://gtrnadb.ucsc.edu/

tRFdb: http://genome.bioch.virginia.edu/trfdb/

MINIbase: http://cm.jefferson.edu/MINTbase/

Omicabean: http://www.omicsbean.cn/

miRBase: http://www.mirbase.org/index.shtml.

piRBase: http://www.regulatoryrna.org/database/piRNA/

### Ischemia model

All institutional and national guidelines for the care and use of laboratory animals were followed. All experiments were approved by the Institutional Animal Care and Use Committee, Capital Medical University, Beijing, China (approved number: AEEI-2015-156). For the mouse ischemia model, 8-week-old C57BL/6 male mice (~20–22 g) were purchased (Biotechnology Co.,Ltd. Beijing, China), and raised in an animal room with 25 °C and a 12 h light/dark cycle. The method for ischemia was followed a previous protocol [22]. A laser Doppler blood flow monitor (RWD, China) was used to ensure successful occlusion (>50% drop from pre-stroke baseline). Sham operation was performed and followed with procedures the same as the ischemic operation. Trial *n* = 4.

### Vibratome sectioning of the mouse brain

After ischemia for one hour, the mice were anesthetized by isoflurane. The heart was perfused with 20 mL of fresh artificial cerebrospinal fluid (ACSF) containing (in mM): 125 NaCl, 5 KCl, 1.2 NaH_2_PO_4_, 26 NaHCO_3_, 1.3 CaCl_2_, 1.3 MgCl_2_, and 10 glucose, pH 7.4, which had been equilibrated with 95% O_2_ and 5% CO_2_. After decapitation, the whole brains were quickly removed and placed in an ice-chilled sucrose slicing solution, which contained the following (in mM): 213 sucrose, 3 KCl, 1 NaH_2_PO_4_, 26 NaHCO_3_, 0.5 CaCl_2_, 5 MgCl_2_, and 10 glucose, pH 7.4. The brain slices (200 μm) were cut with a vibratome (VT1200, Leica, Germany) and recovered in ACSF for at least 1 h before protein synthesis assay.

### Protein synthesis assay

For the primary neuron staining, the nascent protein synthesis was detected by the Global Protein Synthesis Assay Kit (Abcam, catalog#: ab235634, USA), according to the manufacture’s instruction. For the brain slice staining, the brain slices were incubated in the protein label solution for 1 h. Then, the fixative solution was added and incubated at room temperature for 1 h. Next, the brain slices were washed three times in PBS and incubated with the permeabilization buffer overnight at 4 °C. The brain slices were incubated with the reaction cocktail for 2 h at room temperature under darkness; then washed 3 times with wash buffer and proceeded with DNA staining by DAPI. The images were obtained by a Leica SP8 confocal microscope. Trial *n* = 2−4.

### Transmission electron microscopy

The neurons were rinsed twice with a phosphate buffer saline (PBS) and fixed with 2.5% glutaraldehyde in 0.1 M PBS (PBS, pH 7.3) for 10 min at room temperature and overnight at 4 °C. Then, the cells were examined under a transmission electron microscopy (TEM) (JEM-1220, Japan) according to a conventional method.

### Protein extraction

Adult flies with 1−3 day old were heat-shocked at 30 °C for 24 h and recovered at 18 °C for 24 h. Then, ~60 fly heads were collected and homogenized in the lysis buffer (50 mM Tris·HCl, pH 7.5, 150 mM NaCl, 0.25% Nonidet P-40) with protease inhibitor mixture (Roche) and phosphatase inhibitors (1 mM NaVO_3_, 50 mM NaF, 30 mM glycerophosphate, and 1 mM EDTA) for 30 min on the ice. The primary neurons were washed twice in PBS, scraped, and then lysed in the lysis buffer for 30 min on the ice. After centrifugation, the supernatant was collected. Trial *n* = 3.

### Identification and differential analysis of proteomics data

Sequential windowed acquisition of all theoretical fragment ion mass spectra (SWATH-MS), a data-independent acquisition (DIA) method was used to analyze the protein levels change in flies and neurons transfected with tRFs. For the flies, every group has three sample replicates and each sample has three technical replicates. For neurons transfected with tRFs, four independent samples were collected for each treatment. For each sample, 100 μg protein lysate was reduced by 8 mM dithiothreitol at 37 °C for 2.5 h to expose the disulfide bonds. After restoring to room temperature, iodoacetamide with the final concentration of 50 mM was added to protect the exposed disulfide bonds through alkylation for 40 min in the dark. The sample was transferred to an ultrafiltration concentrate tube (Millipore, USA) with the relative molecular weight of 10 000. The ultrafiltration tube was centrifuged at 10,000×*g* at 20 °C for 30 min. Add 200 μL lysis into the ultrafiltration tube and centrifuge it for 10,000×*g* at 20 °C for 30 min. Repeat this step 2−3 times. Then, adding 200 μL 50 mM NH4HCO3 to the ultrafiltration tube and centrifuge it for 10,000×*g* at 4 °C for 30 min. Repeat this step 2−3 times. Add 150 μL 50 mM NH4HCO3 to the inner tube of a new ultrafiltration tube and then added the enzyme solution according to the ratio of protein: trypsin = 50:1, and incubated at 37 °C for 16 h. The samples were centrifuged at 10,000×*g* at 4 °C for 30 min, then 100 μL 50 mM NH4HCO3 was added and centrifuged at 10,000×*g* at 4 °C for 30 min. The procedure was repeated three times to collect the polypeptide samples. Finally, a final concentration of 1% formic acid is added to the sample and the sample is lyophilized.

The enzyme-cut samples were redissolved in A-phase solution (containing 0.1% formic acid and 2% acetonitrile). The samples were quantified by Nanodrop, centrifuged at 18,000×*g* for 10 min, and 4 μL supernatant was extracted for analysis by Liquid Chromatography and Triple TOF 6 600 Mass Spectrometry. Chromatographic column: C18 desalination (3 μm, 120 Å, 350 μm × 0.5 mm), analysis of C18 column (3 μm, 120 Å, 75 μm × 150 mm), column temperature of 40 °C. The system pressure is less than 6 000 Psi. Flow rate: 300 nl/min;1 h analysis gradient: 0 min, 5% B (B phase solution containing 0.1% formic acid and 98% acetonitrile); 0−0.1 min, 5−9% B; 0. 1−32 min, 9−22% B; 32−48 min, 22−32% B; 48−51 min, 32−40% B; 51−51.1 min, 40−80% B; 51.1−56 min, 80% B; 56−56.1 min, 80−5% B; 56.1−60 min, 5% B. Mass spectrum conditions: ESI source, positive ion scanning mode, spray voltage 2.3 kV, ion source temperature 150 °C. The scanning mode of mass spectrometry for protein identification: first stage full scanning, mass to charge (m/z) range of 350−1500; at the second stage, 40 precursor ions with high strength were selected for collision-induced dissociation (CID). Scanning range m/z 100−1500, dynamic exclusion scanning, dynamic exclusion time 10 s. SWATH protein quantitative model: first-stage full scan, m/z range 350−1500. Based on protein identification of liquid phase TiC in m/z 400−1225, a range of 60 variable secondary scanning windows was set. A narrow mass number window was used when the precursor ion was dense to enhance selectivity, and a wide mass number window was used when the precursor ion density was low to cover a wider range of precursor ions. The CID was dynamically fragmented and uniformly scanned. Each *Drosophila* sample was collected three times and each rat neuron sample was collected once.

The Protein identification was done through Protein Pilot Software 5.0 (SCIEX). The Uniprot database was used to set the first-level mass deviation of 10 ppm and the second-level mass deviation of 20 ppm to obtain a spectral library with high confidence (false discovery rate <1%). The retention time of parent ion and fragment ion in the spectrum library established by Data Dependency Analysis (DDA) was extracted with PeakView2. 2 software. The retention time of fragment ion generated by corresponding SWATH was matched to extract the fragment ion chromatographic peaks of corresponding peptides. The strength and retention time integral is used to calculate the peak area. PeakView2.2 software set the following conditions: each protein contains at least 10 peptides and was a unique peptide; The fragment ion (transition) of each peptide was 6. The extraction window was set for 6 min. In different elution time, peptides with better abundance and peak shape were selected to correct the retention time of other peptides. The results contained the ion peak area of the fragment, the peak area of the peptide segment, and the peak area of the assembled corresponding protein. The relative quantity of query peptides among samples was obtained. Markview 1.2 software was used to analyze the difference of data and t-test showed that the difference of protein.

DIA was used to detect the protein expression profile of the neurons with different treatments including vehicle, glutamate, glutamate + Exp pep, glutamate + DM pep. Trial *n* = 3. Using high-PH reversed-phase chromatography, we pre-fractionated the peptides for the DDA analysis. Fifty micrograms of peptides have a sequential elution with 9, 12, 15, 18, 21, 25, 30, and 50% acetonitrile in ammonia water, pH 10. All fractions were collected, heat-dried, and stored at −80 °C after centrifugation. The LC-MS/MS system contains a nanoflow high-performance liquid chromatograph instrument (Easy-nLC 1 000 System, USA) coupled with a Q-Exactive HF mass spectrometer (USA). The peptide was separated with a 30 cm of self-packing column (150 μm inner diameter, ReproSil-Pur C18-AQ, 1.9 μm, Germany) at 60 °C. Flow rate: 500 nL/min. 135 min analysis gradient: 0−13 min, 6−10% B; 13−99 min, 10−23% B; 99−120 min, 23−33% B; 120−123 min, 33−90% B; 123−135 min, 90% B. The scan range was 375~1 400 Da. According to the manufacturer’s instructions, iRT (Biognosys, Schlieren, Switzerland) was used as an internal standard.

For the DIA analysis, the LC-MS/MS system contains a nanoflow HPLC (Easy-nLC 1 200 System; Thermo Fisher) coupled to a Q-Exactive HF mass spectrometer (Thermo Fisher). The wide MS1 scan range: 400−1 200 m/z; resolution:120,000; AGC: Standard; RF lens (%): 30; maximum injection time mode: custom; maximum injection time (ms): 50. The narrow MS2 scan range: 400−800 m/z (20 × 20 m/z), 800−1000 m/z (5 × 40 m/z), 1000–1200 m/z (4 × 50 m/z); resolution:60,000. The raw data of proteomics were matched with the Uniport database by the software v1.6.2.3 (http://www.maxquant.org/). The false discovery for both proteins and peptides was set 1%. We analyzed the DDA and DIA data with Spectronaut^TM^ (v13.9). Then, the peptides were further evaluated by the Perseus software v1.6.1.3 (http://www.perseus-framework.org; Max Planck Institute of Biochemistry, Germany). The cut-off values for upregulated and downregulated ratio at >1.5, with *P* value set at 0.05.

### Quantitative real-time PCR

Total RNAs were collected by Trizol treatment (Invitrogen, USA) from the flies. Then, the RNAs (2 μg) were reverse transcribed with the Revert Aid First Strand cDNA Synthesis kit (Thermo Scientific, USA). The total volume of q-PCR was 10 μL using PowerUp^TM^ SYBR^TM^Green master mix [2X] (A25741, ThermoFisher) 5 μL, forward primer 0.5 μL, reverse primer 0.5 μL, and DNA template + Nuclease-free water 4 μL. The q-PCR was performed in triplicate using the QuantStudio 6 system. The quantification of the target gene was performed by ΔΔCt method. Actin5C was set as a reference of the total RNA quantity. To determine the *GluR1*^*Lc*^ expression level, two primer sets were used. The average of the two primer sets was analyzed and plotted. Trial *n* = 3. The following primers were used:

*Ago1*:

Forward: 5- GCCCGGCAAGACTCCAGTA -3

Reverse: 5- CCCAGAACGGTGTCACCTACA -3;

*Ago2*:

Forward: 5- TGCCACATGTTCCCTCGTT -3;

Reverse: 5- AGGCCGGAGCCGGATA -3;

*Actin5c*:

Forward: 5- CGAGGCCCCGCTGAAC -3

Reverse: 5- TCGAACATGATCTGGGTCATCT -3;

*GluR1*^*Lc*^:

Forward-1: 5- CGCCTCAACGCCATCCT -3

Reverse-1: 5- GGTACCCGATGCCGTTCTTT -3;

Forward-2: 5- GGCGCCGGACCAACTAC -3

Reverse-2: 5- AATCTTGCGGATTCCATCATG -3

### High-content cell analysis

The primary neuron cultures were pretreated with 4 μM Exp pep or DM pep for 1 h and then treated with 200 μM glutamate for 30 min. Protein synthesis assay was performed as the protocol mentioned above. Multichannel fluorescence scanning was performed by the high-content analyzer (ArrayScan XTI, Thermo Scientific, USA). In the 24-well plate, each well was scanned for 25 times, and the average fluorescence intensity was calculated by the instrument.

### tRFs transfection

The cells were plated with a density of 3.5 × 10^5^ per well in a 24-well dish. Then, tRFs or piRNAs were transfected into the primary neurons at day 4 or 5. Transfection reagent (0.8 μL) Lipo8000™ (Invitrogen, USA) was added into a 25 μL neurobasal medium without serum and Penicillin-Streptomycin (Invitrogen, USA). Next, tRFs (1.25 μM) were added into the above solution and incubated for 20 min at room temperature. Then, the solution was transfected into the primary neuron cultures. The synthetic tRFs or piRNAs were used:

piR-rno-318194: AGTTTAAGTAGAATATCAGCTTTGG;

piR-rno-888357: GTTTAAGTAGAATATCAGCTTTGG;

piR-rno-2898628: AAATTTTCGTAGGTTTGAATCCTT;

tRF-Trp-TCA-002: AACAAGTTTAACTTCTGCCA;

tRF-Pro-TGG-016: CAAGAAGTAGTTTAAATAGAATATCAGC;

tRF-Tyr-GTA-038: TTGAGCCCCCTTTTTACCACCA;

tRF-Gln-CTG-021: UGGACUCUGAAUCC;

tiRF-Ser-GCT-001: AAGAAAGUAUGCAAGAACUG;

tRF-His-GTG-017: TAGACTGTGAATCT;

tRFs-random: UCGUGCCCAGGCAC

### The synthesis of peptides

- Exp pep: YGRKKRRQRRRGSARAEVHLRKS;
- DM pep: YGRKKRRQRRRGSAAAEVHLAKS.
- The peptides (purity >98%) were synthesized in GL Biochem Ltd. (Shanghai, China).

### Statistical analyses

For experiments to quantify fly behavior and for sample collections, the experimenters were blinded to the genotype and interventions; the studies were conducted by two different experimenters. The number of animals studied was based on our previous experience and preliminary investigations. We did not exclude any data from this study. Statistical analysis was performed with an unpaired *t*-test between two groups. For comparison of more than two groups, one-way ANOVA was applied, with post hoc analysis *Tukey* for comparison between two groups. Relative risk is set with odds ratios 95% confidence interval, mean ± SD is shown. A *P* < 0.05 is considered significant. * *P* < 0.05, ** *P* < 0.01, and *** *P* < 0.001.

## Result

### The expression profile of small non-coding RNAs was altered in necrosis of primary neuron cultures

**Fig. 1:**
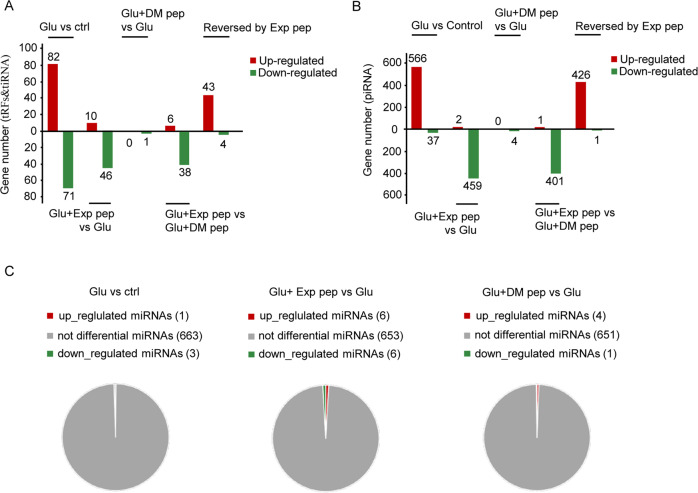
Profile of non-coding RNAs in neuronal necrosis. The rat embryonic primary neuron cultures were treated with the following conditions: control (no treatment), glutamate treatment, treatment with glutamate and Exp pep, and treatment with glutamate and DM pep. Then, total RNAs were collected, sequenced, and analyzed accordingly. The number of changes of different RNA species were compared for the treatment condition indicated on the graph. tRFs & tiRNAs (**A**), piRNAs (**B**), and miRNAs (**C**) were listed separately. The Y axes are the upregulated or downregulated number of transcripts, indicated by the red column and green column, respectively. The change of more than 50% is set as the cutoff with *P-*value < 0.05. Three independent samples were collected for each treatment. These three samples were treated as a group to compare between groups.

In primary embryonic rat cortical neuron cultures, the addition of 200 μM glutamate (glu) in the medium for 4 h could induce necrosis in nearly 95% of neurons; at 30 min after glu treatment, the increase of H3K4me3 was observed, indicating it is an early response of the cellular insult [5]. We focused on the small non-coding RNAs, because small RNAs such as tRF^Tyr-GTA^ and tiRF^tyr^ are toxic to neurons [16, 23]. Using a high-throughput RNA sequencing, we analyzed the enrichment of tRFs, piRNAs, and miRNAs in the primary neuron cultures treated with glu. The nucleotide length distribution of tRFs, piRNAs, and miRNAs were compared for the following conditions: control (no glu treated), glu (with glu treatment for 30 min, induce necrosis), Exp pep + glu (with Exp pep pretreated for 1 h followed by glu treatment for 30 min, rescue necrosis) and DM pep + glu (with DM pep pretreated for 1 h followed by glu treatment for 30 min, no rescue of necrosis) (Fig. S1). This data suggests the overall length distribution of the sequenced ncRNAs were unaltered during neuronal necrosis, indicating either few ncRNAs were enriched or H3K4me3-dependent transcription was unbiased for length. Consistent with fewer ncRNAs to be enriched, compared the glu treated group with the control group (glu/control), 82 tRFs & tiRNA were upregulated (fold change > 1.5, *P* < 0.05), and 71 tRFs & tiRNA were downregulated (fold change > 1.5, *P* < 0.05) (Fig. 1A and Tables S1, 2). Compared Exp pep + glu/glu, 10 tRFs & tiRNA were upregulated and 46 tRFs & tiRNA were downregulated (Fig. 1A and Tables S3, 4). Only one tRFs was downregulated comparing the DM pep + glu/glu (Fig. 1A and Tables S5, 6). Compared the Exp pep + glu with the DM pep + glu, 6 tRFs & tiRNA were upregulated, and 38 tRFs & tiRNA were downregulated (Fig. 1A and Tables S7, 8). The shared tRFs & tiRNA, which were upregulated in glu/control but downregulated in Exp pep + glu/glu are likely transcriptional activated mediated by the increased H3K4me3 (Table S9). For the piRNA, compared the glu/control, 566 piRNAs were upregulated (fold change > 2, *P* < 0.05), and 37 piRNAs were downregulated (fold change > 2, *P* < 0.05) (Fig. 1B and Tables S10, 11). Compared Exp pep + glu/glu, two piRNAs were upregulated and 459 piRNAs were downregulated (Fig. 1B and Tables S12, 13). Four piRNAs were downregulated comparing the DM pep + glu vs glu (Fig. 1B and Tables S14, 15). Compared Exp pep + glu/DM pep + glu, 1 piRNA was upregulated and 401 piRNAs were downregulated (Fig. 1B and Tables S16, 17). Moreover, the shared piRNAs which were upregulated in glu/control but downregulated in Exp pep + glu/glu are likely transcriptionally activated mediated by the increased H3K4me3 (Table S18). In general, miRNAs showed less changes under the different conditions (Fig. 1C), indicating that certain ncRNAs (such as tRFs and piRNAs) are likely transcriptionally activated during neuronal necrosis.

### Recombinant tRFs & tiRNA were toxic to neuron culture

**Fig. 2:**
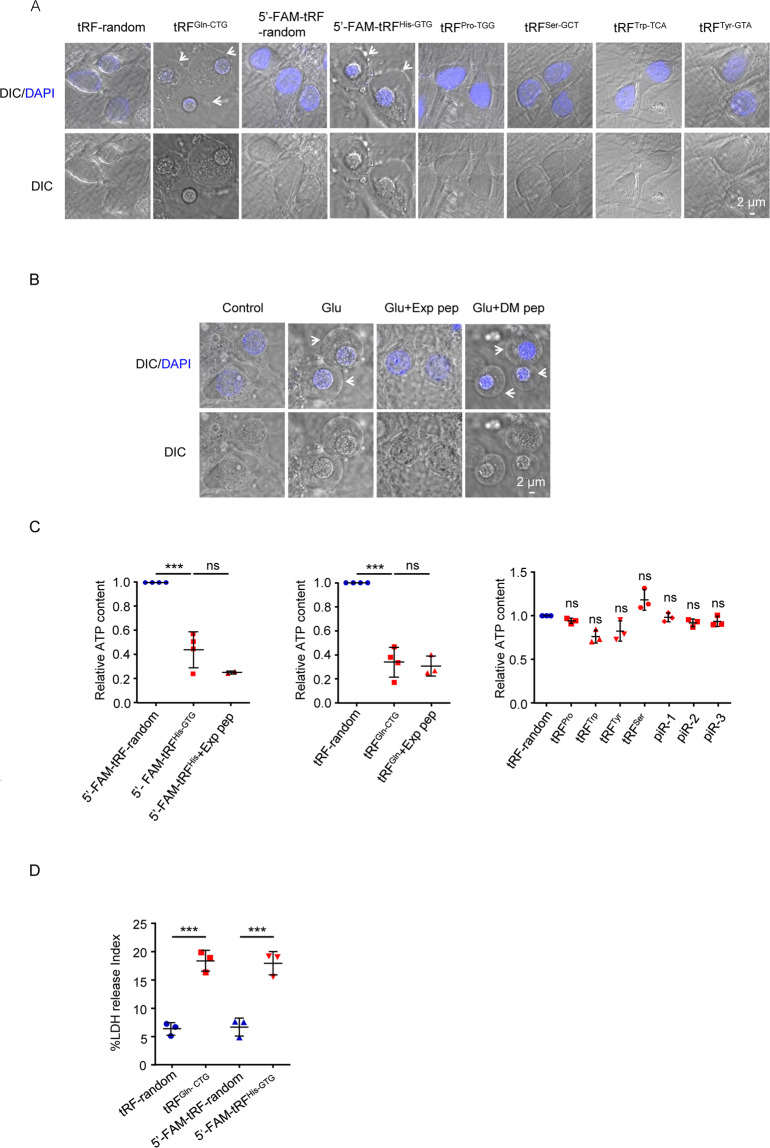
The cytotoxic effect of transfected tRFs and piRNAs in neuronal culture. Synthetic tRFs and piRNAs were transfected in primary neuron culture. For controls, a scrambled sequence of tRF was synthesized (tRF-random), or the FAM tag was added at the 5′ of the control (5′-FAM-tRF-random). Then, their toxicity was assessed by the ATP content and LDH release assays. **A** After 4 h of tRFs transfection, typical morphological change of neurons under confocal. White arrows pointed to the swollen neurons. Trial *n* = 3. **B** The swollen neuron morphology was also observed after glutamate treatment for 3 h. Trial *n* = 3. **C** After 3 h transfection, the total cellular ATP level was determined. Three synthesized piRNAs were tested, including piR-rno-318194 (piR-1), piR-rno-888357 (piR-2), and piR-rno-2898628 (piR-3). Because of variations in each culture condition (different embryo age and cell number), the ATP levels of the controls were set as 1, and relative ATP level changes of the other conditions (tRFs and piRNAs) were plotted. Trial *n* = 2−4. One-way ANONA analysis was performed for comparison of more than three groups in all of the following figures, with post-hoc test *Tukey* for comparison between two groups. * *P* < 0.05, ** *P* < 0.01, and *** *P* < 0.001. **D** For the LDH assay to quantify cell death, the cell medium and neurons were collected separately at 12 h after transfection. The cell death index = LDH_medium_/ (LDH_medium_ + LDH_cell_) × 100. The conditions of treatment were listed on the dot graph. Trial *n* = 3.

To test whether the enriched tRFs & tiRNA or piRNAs were cytotoxic, we synthesized six tRFs & tiRNA and three piRNAs, which were upregulated in the glutamate treated group and reversed by the Exp pep. These ncRNAs were synthesized and individually transfected into the rat cortical neuron cultures with a concentration range 0.5−1.25 μM, which is lower than the physiological concentration of tRNAs (10−200 μM) [9]. To determine whether a given synthetic tRFs & tiRNA could enter neurons, FAM, a green fluorescent dye, was covalently added to the 5′ end of tRNA-derived fragments (5′-FAM-tRF^His-GTG^, belongs to the subclass tRF-2), with the random tRF^His-GTG^ sequence as a control (5′-FAM-tRF-random). After transfection for 30 min, the FAM fluorescence was detectable (Fig. S2). Next, we tested several tRFs without a tag, including tRF^Gln-CTG^ (tRF-2), tRF^Trp-TCA^ (tRF-3b), tRF^Pro-TGG^ (tRF-5c), tiRNA^Ser-GCT^ (tiRNA-5), and tRF^Tyr-GTA^ (tRF-3b). Four hours after transfection, 5′-FAM-tRF^His-GTG^ and tRF^Gln-CTG^ induced neurons to swell and subsequently plasma membrane rupture with the nucleus becoming smaller; while tRF^Trp-TCA^, tRF^Pro-TGG^, tiRNA^Ser-GCT^, and tRF^Tyr-GTA^ showed no such toxicity (Fig. 2A). Waited for 12 h or longer, tRF^Pro-TGG^ and tRF^Tyr-GTA^ also showed mild cytotoxicity. The neuron swelling induced by the tRFs was similar as neurons received glutamate treatment (Fig. 2B). The cell death was quantified by the ATP assay. 5′-FAM-tRF^His-GTG^ or tRF^Gln-CTG^ treatment resulted in declined cellular ATP level; whereas tRF^Trp-TCA^, tRF^Pro-TGG^, tRF^Tyr-GTA^, tiRNA^Ser-GCT^, and piRNAs had no effect (Fig. 2C). To further confirm the cytotoxicity, the LDH release assay showed a similar result (Fig. 2D). The cell death induced by tRF^Gln-CTG^ belonged to necrosis (Fig. S3). Further, the Exp pep showed no rescue against 5′-FAM-tRF^His-GTG^ or tRF^Gln-CTG^ cytotoxicity (Fig. 2C), indicating these tRFs functioning at the downstream of H3K4me3 increase. Together, these results indicate that the enrichment of tRFs may be the inducer of the neuronal necrosis.

### tRFs reduced nascent protein synthesis during neuronal necrosis

**Fig. 3:**
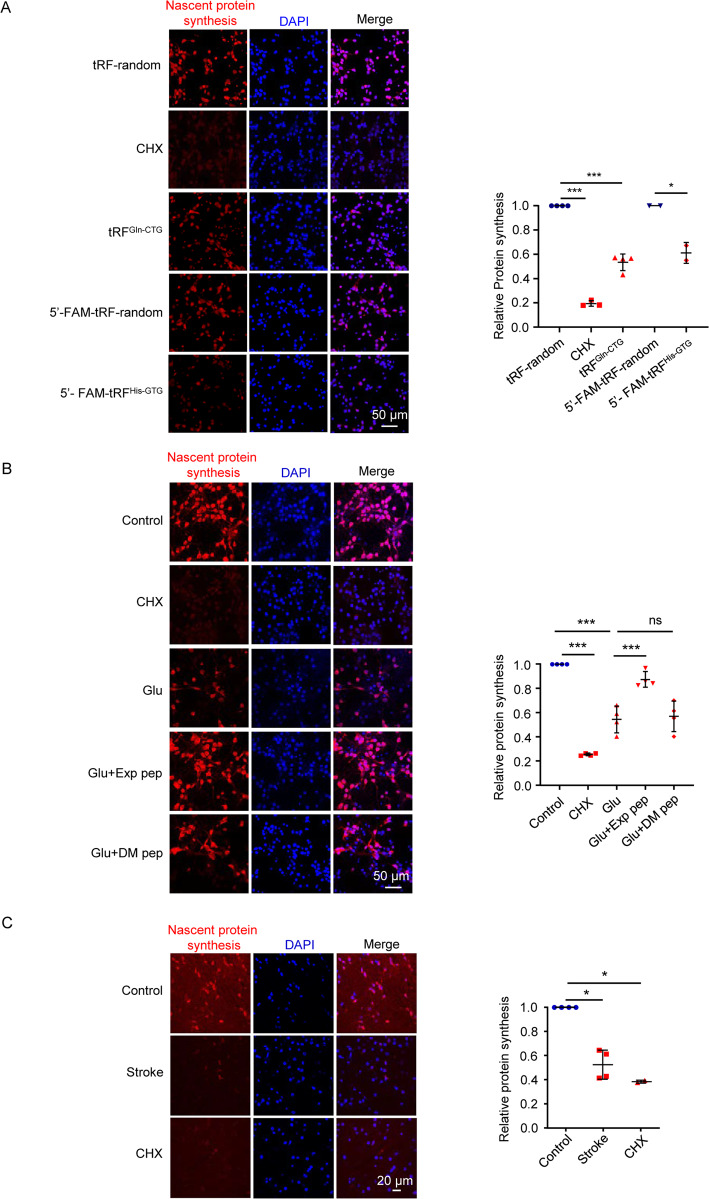
Nascent protein synthesis during neuronal necrosis. **A** After transfection of tRFs for 1 h in primary neurons, the nascent protein synthesis was determined by a kit. Cycloheximide (CHX) is a well-known protein synthesis inhibitor with 10 μM applied. For each treatment, five fields were randomly selected. The averaged fluorescent intensity of the control was set as 1, and the relative intensity of the experimental groups was determined and plotted (the same quantification method was used in (**B**, **C**). Trial *n* = 4. *** *P* < 0.001. **B** After glutamate treatment for 30 min, nascent protein synthesis was determined, with or without Exp pep or DM pep pretreatment. Trial *n* = 4. **C** Alive brain slices were obtained 1 h after an ischemic stroke model in mice. As a control, brain slices from the sham-operated mouse were obtained. The sham-operated mice were also treated with CHX for 1 h, which served as a positive control for the inhibition of protein synthesis. Then, the protein synthesis assay was performed in the brain slices. For the ischemia model and sham control, four mice were tested, respectively. Two mice were tested for the CHX treatment.

Given that certain tRF species can reduce protein translation through competing mRNA binding on ribosome or affecting the assembly of the translation initiation machinery or disrupting the peptidyl transferase activity on the ribosome [9, 24–26], we tested whether 5′-FAM-tRF^His-GTG^ and tRF^Gln-CTG^ might affect protein synthesis, which was detectable by the Global Protein Synthesis Assay Kit [27]. This kit utilizes the “OP-puro” probe, a cell-permeable alkyne containing puromycin analog, which stops translation by forming covalent conjugates with nascent polypeptide chains. Then, the truncated peptides may be rapidly turned over by the proteasome to be detectable by the fluorescent azide. Before cell swelling, we observed that protein synthesis was already declined (Fig. 3A, B); treatment with the Exp pep rescued the reduction of protein synthesis induced by glutamate (Fig. 3B). This result was further confirmed by a high-content analysis, which quantifies the fluorescent intensity by the instrument in an unbiased manner (Fig. S4). In a mouse model of ischemic stroke, the declined nascent protein synthesis was also observed before brain damage occurred (Fig. 3C and Fig. S5). Together, these results suggest that tRFs may trigger the reduction of protein synthesis in the early phase of neuronal necrosis.

### Ribosomal stalling may explain the reduced protein synthesis during neuronal necrosis

**Fig. 4:**
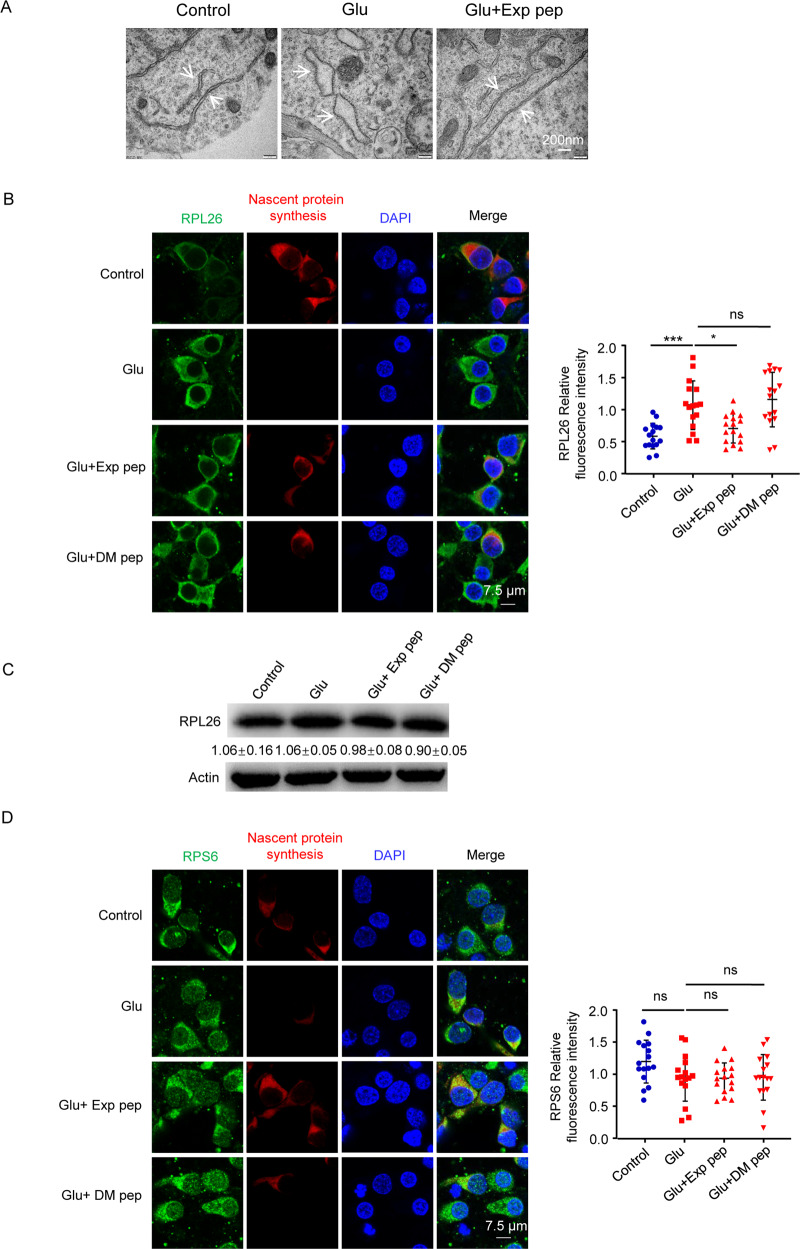
Ribosomal staining pattern in neuronal necrosis. **A** Representative morphology of ribosome imaged by the transmission electron microscopy. Primary neurons were treated with glutamate for 30 min with or without pre-treatment of Exp pep. White arrows point to the endoplasmic reticulum. **B** Representative RPL26 immunostaining pattern in neurons treated with glutamate, and with or without Exp pep or DM pep pretreatment. Eight random selected fields were quantified for each trial. Trial *n* = 2. The fluorescent intensity of each field was plotted. **C** With or without Exp pep or DM pep pretreatment, primary neurons were treated with glutamate for 30 min. Then, total proteins were collected and the protein level of RPL26 was determined by Western blot. Trial *n* = 3. **D** With the same condition as in (**B**), the representative RPS6 immunostaining pattern in neurons. Five random selected fields were quantified for each trial. Trial *n* = 3.

To test how the cytotoxic tRFs may induce suppression of protein synthesis, we examined ribosomal stalling. Using a transmission electron microscope, we observed that the number of ribosomes on the endoplasmic reticulum (ER) was increased upon glutamate treatment, and abolished by the Exp pep (Fig. 4A). The increased number of ribosomes on ER is likely due to failure of ribosome disassembly. In addition, ER morphology appeared expanded upon glutamate treatment. Consistent with this, ER stress is known to induce ER expansion in photoreceptor cells [28]. Immunostaining the 60S ribosomal protein L26 (RPL26) and 40S ribosomal protein S6 (RPS6), the core component of large and small ribosomal subunit protein, we observed that the reduced nascent protein synthesis in glutamate-induced neuronal necrosis was associated with an enhanced staining of RPL26; this phenomenon could be abolished by Exp pep (Fig. 4B and Fig. S6A); whereas the total protein level of RPL26 was unaltered in these cells (Fig. 4C). Interestingly, the staining intensity of RPS6 was not changed during necrosis (Fig. 4D and Fig. S6B). The increased RPL26 fluorescence intensity is likely due to less turnover of the large ribosomal subunit during protein synthesis. Alternatively, ER stress/expansion may affect the appearance of ribosomes on ER [28]. This phenomenon is consistent with the ribosomal quality control process, which initially removes the small subunit of the stalled ribosomal complex for a new cycle of protein translation [29].

### Proteins function in mitochondria might be the target of the cytotoxic tRFs

To determine the affected pathways by tRFs, we selected eleven tRFs that were enriched in necrosis and significantly rescued by Exp pep (Table S19). We predicted their mRNA targets by a base-pairing method, which requires the predicted RNA sites to satisfy a threshold of the algorithm (miRanda and TargetScan) [30, 31]. The target genes were listed (Tables S20−30). Then, the enriched biological process and pathways were evaluated by the Gene Ontology (GO) and Kyoto Encyclopedia of Genes and Genomes (KEGG) methods [32, 33]. The result showed that genes involved in the metabolic process were enriched, with the peroxisome proliferator-activated receptor (PPAR) pathway mostly altered (Fig. S7 and Tables S31, 32). PPAR is known to regulate metabolism by affecting mitochondrial respiratory chain complex IV [34]. The enriched peroxisome pathway is also known to regulate mitochondrial function, such as β-Oxidation of fatty acids and reactive oxygen species metabolism [35]. Similarly, the other enriched pathways are also known to involve mitochondrial functions, including the insulin resistance pathway, circadian clock, butanoate pathway, adipocytokines, and metabolism of ketone bodies [36–41]. In general, these tRFs affected genes and pathways seem to enrich in the mitochondrial metabolism.

**Fig. 5:**
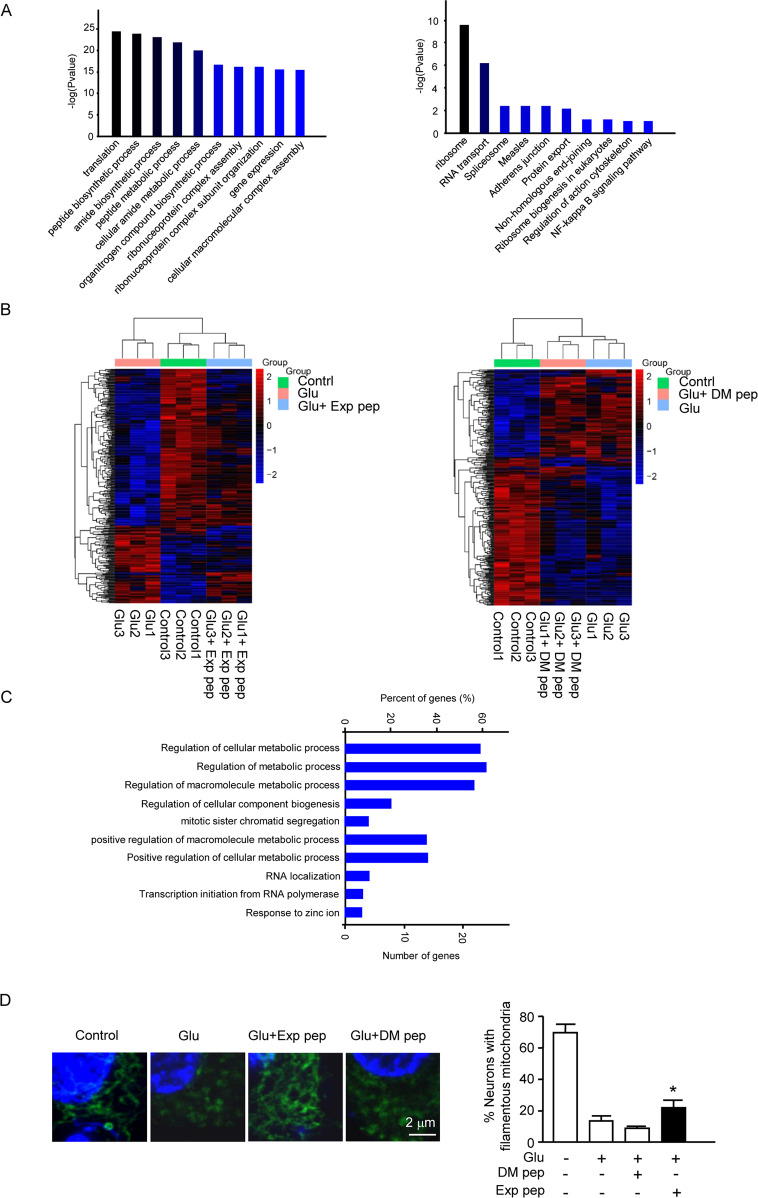
The proteomic analysis to determine tRF and glutamate effect on metabolism. **A** One hour after transfection of the cytotoxic tRF, total proteins were collected and analyzed by proteomics. The results of GO and KEGG analysis were shown. Four independent samples were collected for each treatment. These samples were treated as a group to compare between groups. The enriched biological processes and pathways are indicated on the graph. **B** Three hours after glutamate treatment with or without Exp pep or DM pep pretreatment, total proteins were collected and analyzed by proteomics. Trial *n* = 3. The heat map showed reduced protein level after glutamate treatment, and Exp pep (but not DM pep) rescued the protein level reduction. **C** The result of the GO analysis was showed for the downregulated protein after glutamate treatment. **D** Mitochondrial fragmentation in neuronal necrosis. Forty minutes after glutamate treatment with or without pretreatment of Exp pep or DM pep, the primary neurons were fixed and immune-stained with TOM20 to label mitochondria. The number of neurons displayed with fragmented mitochondria were determined. Trial *n* = 4. For each treatment, 15 randomly selected fields were examined for each trial.

To validate the above analysis, mass spectrometry was performed to compare the tRF^Gln-CTG^ treated group to the control group; and 113 downregulated and 42 upregulated proteins were detected (fold change > 1.5, *P* < 0.05) (Table S33). The GO and KEGG analysis of downregulated proteins revealed that the translational, metabolic biological process, and ribosome pathway were significantly affected (Fig. 5A and Tables S34, 35). This further demonstrated that the protein synthesis and ribosomal pathway were likely disrupted in neuronal necrosis.

To compare the affected pathways of tRFs after glutamate treatment, total proteins were collected and analyzed by mass spectrometry, with the same amount of protein loaded for analysis in each sample. Compared the glutamate treated group to the control group, 21 proteins were upregulated (fold change > 1.5, *P* < 0.05), and 105 proteins were downregulated (fold change > 1.5, *P* < 0.05) (Table S36). Compared the Exp pep + glu/DM pep + glu, 18 proteins were upregulated (fold change > 1.5, *P* < 0.05), and 19 were downregulated (fold change > 1.5, *P* < 0.05) (Table S37). The reduced level of proteins identified in the glu/control could be rescued mostly by Exp pep, but not DM pep (Fig. 5B and Tables S38, 39). The GO analysis for the downregulated proteins (glu/control) revealed that the metabolic process was significantly affected (Fig. 5C and Table S40). This result is consistent with the analysis of the tRFs target genes. Downregulated proteins included the nuclear receptor coactivator, ubiquitin-associated protein 2, galectin, and oxysterol-binding protein; and these proteins are known to regulate the dynamic control of mitochondrial biogenesis, energy metabolism, mitochondrial size, and respiration [42–45], indicating that mitochondrial function may be compromised.

To determine whether mitochondrial impairment functions downstream of tRFs enrichment, we examined the mitochondrial morphology in necrotic neurons. The result showed that mitochondrial fragmentation appeared upon glutamate treatment and could be rescued by Exp pep, but not DM pep (Fig. 5D). Further, a dynamin-related protein 1 (Drp1) inhibitor, mdivi-1, could rescue the neuronal necrosis (Fig. S8).

### Small RNA transcription, transport, and short-interfering-RNA-mediated gene silencing played functional roles in the *Drosophila* model of neuronal necrosis

**Fig. 6:**
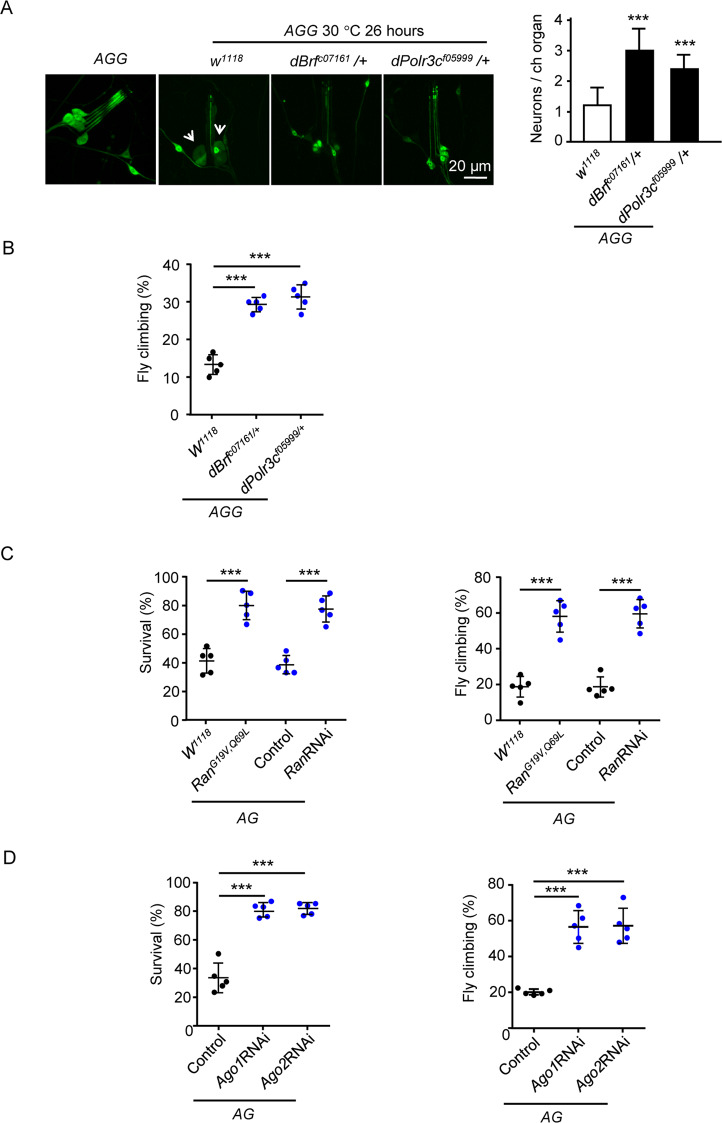
The *Drosophila* model of neuronal necrosis. **A** Effect of mutations in the *RNA polymerase III* complex on neuronal necrosis. The images are samples of one chordotonal organ neuron (Ch N), which contains six sensory neurons in a cluster. Compared to the wild-type flies, the activation of neuronal necrosis resulted in Ch N loss in the *AGG* (*Appl-Gal4*/+; *UAS-GluR1*^*Lc*^, *tubulin-Gal80*^*ts*^/+; *UAS-GFP*/+) flies. The white arrows point to the swollen neurons. In the mutants under the *AGG* background (*Appl-Gal4*/+; *UAS-GluR1*^*Lc*^, *tubulin-Gal80*^*ts*^/+; *UAS-GFP*/*dBrf*^*c07161*^ and *Appl-Gal4*/+; *UAS-GluR1*^*Lc*^, *tubulin-Gal80*^*ts*^/+; *UAS-GFP*/*dPolr3c*^*f05999*^), the average number of Ch N was plotted. *n* = 25 chordotonal organs from five larvae. **B** The climbing ability of *AGG* flies under the mutant background same as (**A**). Trial *n* = 5, 50−60 flies were tested for each trial. **C** The survival and climbing ability of *AG* (genotype: *Appl-Gal4*/+; *UAS-GluR1*^*Lc*^, *tubulin-Gal80*^*ts*^/+; *+*/+) flies under the mutant or RNAi background (genotype: *Appl-Gal4*/+; *UAS-GluR1*^*Lc*^, *tubulin-Gal80*^*ts*^/*Ran*^*G19V,Q69L*^;+/+ and *Appl-Gal4*/+; *UAS-GluR1*^*Lc*^, *tubulin-Gal80*^*ts*^/*+*; *UAS-RanRNAi* /+). Besides the control under the mutant background (*w*^*1118*^), a genetic background matched RNAi control (TB00072) was also used. This line contains an insert of the transgenic vector into the defined site on chromosome 3. Trial *n* = 5, 50−60 flies were tested for each trial. **D** The survival and climbing ability of *AG* flies under the *Ago1RNAi* and *Ago2RNAi* background (genotype: *Appl-Gal4*/+; *UAS-GluR1*^*Lc*^, *tubulin-Gal80*^*ts*^/*UAS-Ago1RNAi*; +/+ and *Appl-Gal4*/+; *UAS-GluR1*^*Lc*^, *tubulin-Gal80*^*ts*^/*UAS-Ago2RNAi*; +/+). A genetic background matched RNAi control (TB00073) was also used. This line contains an insert of the transgenic vector into the defined site on chromosome 2. Trial *n* = 5, 50−60 flies were tested for each trial.

Previously, we established a genetic model of neuronal necrosis in *Drosophila*, which contains three components: the neuron-specific promoter *Appl-Gal4*, the transgenic rat glutamate receptor 1 Lurcher mutant (a constitutively opened cation channel) *UAS-GluR1*^*Lc*^, and a ubiquitously expressed *tubulin* promoter driven by the temperature-sensitive *Gal80* (a *Gal4* inhibitor) *tubulin-Gal80*^*ts*^. The model system *Appl* *>* *GluR1*^*Lc*^*; tubulin-Gal80*^*ts*^ is simplified as the *AG* flies [5]. At permissive 18 °C, the *AG* flies developed normally. Upon shifting the temperature to 30 °C for more than 12 h, the inhibitory effect of *Gal80*^*ts*^ on *Gal4* was abolished, resulted in the expression of *GluR1*^*Lc*^, which induced intracellular calcium overload and neuronal necrosis. Due to the difficulty to conduct the genetic manipulations to overexpress or knockout tRFs, we tested the genetic manipulation of the *RNA polymerase III*, which is responsible for the transcription of ncRNAs [46]. Neurons were labeled by a GFP transgene under the *AG* background (*Appl* *>* *GluR1*^*Lc*^*/GFP; tubulin-Gal80*^*ts*^ line, simplified as *AGG*) [5]. After incubation at 30 °C for 26 h, the larval chordotonal neurons (Ch N) showed swollen soma and dendritic segments (Fig. 6A). Under the *dBrf*^*c07161*^ or *dPolr3c*^*f05999*^ (mutations of *RNA polymerase III* subunits) background, the neuronal necrosis and climbing ability were rescued (Fig. 6A, B), suggesting the transcription of ncRNAs to be detrimental in the *AG* flies. Next, we test the role of RNA transport, which is regulated by RanGTPase [47]. The result showed that *Ran*^*G19V,Q69L*^ or *RanRNAi* both rescued the *AG* flies (Fig. 6C). Upon ncRNAs enter the cytoplasm, they can induce the RISC effects through Ago proteins [48, 49]. We found that RNAi of *Ago1* and *Ago2* increased the survival and climbing ability of the *AG* flies (Fig. 6D and Fig. S9). This result suggests that some ncRNAs play a detrimental role in neuronal necrosis depending on the RISC pathway. Because *Ago1RNAi*, *Ago2RNAi*, *RanRNAi*, and *Ran*^*G19V,Q69L*^ did not decrease the *GluR1*^*Lc*^ expression level after a 30 °C heat shock (Fig. S10), the rescue of *AG* flies by these lines should be downstream of the *GluR1*^*Lc*^ expression. Collectively, the studies in the *AG* flies suggest that transcription, transport, and Ago-dependent gene silencing of certain ncRNA species, play a detrimental role in neuronal necrosis.

### Relative protein levels functioning in ribosome and mitochondria were declined in the *AG* flies

**Fig. 7:**
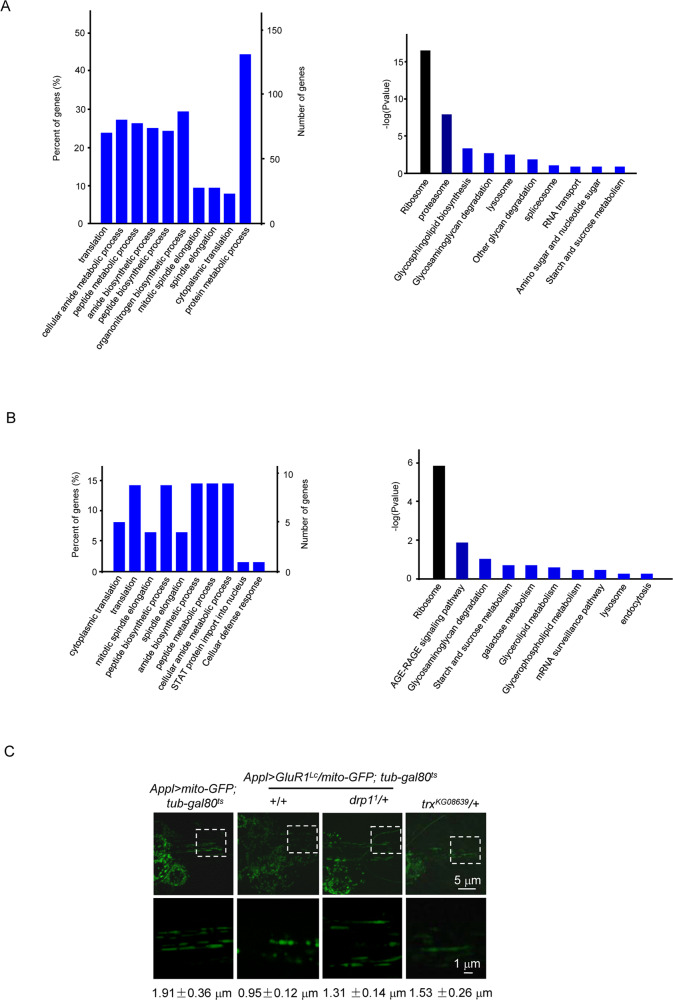
Proteomic analysis of the *AG* flies. Proteomic analysis was performed before and after the induction of neuronal necrosis in the *AG* flies. **A** Compared to the control (*AG* flies without induction of necrosis), the top 10 enriched GO annotations and KEGG pathway analysis of downregulated proteins are listed. Three independent samples were collected and analyzed as a group. **B** Characterization of the proteomic profile of *Ago2RNAi* in the *AG* fly. Two protein profiles were obtained. One was the “necrotic profile”, which was obtained by comparison of the necrotic *AG* flies with the *AG* flies without induction of necrosis. The other one was “*Ago2RNAi* rescue of necrotic profile”, which was obtained by comparison of *AG* (induced necrosis) vs *AG* (induced necrosis under the *Ago2RNAi* background). Compared the two protein profiles, the reversed protein profile of *Ago2RNAi* against the “necrotic profile” was performed the GO and KEGG pathway analysis. **C** The morphological change of mitochondria in the *AG* flies. To label mitochondria, the *UAS-mitoGFP* line was crossed into the *AG* background. At 30 °C, the mitochondria of neurons in the wild type flies *Appl* *>* *mitoGFP*; *tub-gal80*^*ts*^ (genotype: *Appl-Gal4/+*; *tubulin-gal80*^*ts*^*/+*; *UAS-mitoGFP/+*) displays a typical morphology with elongated shape in the Ch N. In contrast, activation of neuronal necrosis resulted in fragmentation of mitochondria in the *AG* (genotype: *Appl-Gal4*/+; *UAS-GluR1*^*Lc*^, *tubulin-Gal80*^*ts*^/+; *UAS-mitoGFP*/+) flies. The mutant *drp1* (*drp1*^*1*^) (genotype: *Appl-Gal4*/+; *UAS-GluR1*^*Lc*^, *tubulin-Gal80*^*ts*^/*drp1*^*1*^; *UAS-mitoGFP*/+) or *trx* (*trx*^*KG08639*^) (genotype: *Appl-Gal4*/+; *UAS-GluR1*^*Lc*^, *tubulin-Gal80*^*ts*^/+; *UAS-mitoGFP*/*trx*^*KG08639*^) rescued the mitochondrial defect. For each genotype, 6−10 Ch N were examined. The mitochondrial length was determined for each genotype of flies as listed. Mean ± SD is shown.

To quantify the protein level changes in the *AG* flies, we performed proteomic analysis to compare the *AG* flies at 18 and 30 °C. 162 proteins were upregulated and 330 proteins were downregulated after the neuron necrosis was induced (fold change > 1.5, *P* < 0.05) (Table S41). In the downregulated category, the translational process was mostly affected (Fig. 7A and Table S42). The downregulated proteins included sulfiredoxin, peroxidase, phosphoenolpyruvate carboxykinase, and E3 ubiquitin-protein ligase parkin. These proteins are known to protect against oxidative damage [50–53]. The KEGG analysis showed that the ribosome signaling pathway was mostly affected (Fig. 7A and Table S43). Interestingly, in the *AG* flies under the *Ago*2*RNAi* background, 345 proteins were differentially expressed after a 30 °C treatment (fold change > 1.2, *P* < 0.05) (Table S44). 69 of 492 differential regulated proteins were reversed by *Ago*2*RNAi* (Table S45). The result of GO and KEGG analysis showed that the reversed protein enriched in the translation and ribosomal pathways (Fig. 7B and Tables S46, 47). This result is consistent with the enrichment of downregulated protein in the necrotic *AG* flies, indicating some ncRNA species function through the Ago pathway to disrupt protein translation and ribosomal pathways.

Further, we found that mitochondrial fragmentation occurred in the *AG* flies (Fig. 7C). Under the background of *drp1* mutant (*drp1*^*1*^), a key regulator of mitochondrial fission, the mitochondrial fragmentation was rescued in the *AG* flies (Fig. 7C). Importantly, suppression of H3K4me3 increase by a trx mutant (*trx*^*KG08639*^) rescued the mitochondrial fragmentation (Fig. 7C), suggesting mitochondrial fragmentation occurred downstream of the H3K4me3 increase.

## Discussion

During neuronal necrosis, abnormal small RNAs may be increasingly transcribed. Our RNAseq data suggested that certain small ncRNAs (14−35 nucleotides) especially the tRFs and piRNAs were enriched in the mammalian neuron necrosis model (rat neuron culture treated with glutamate). This enrichment could be reversed by the Exp pep, an interference peptide to disrupt the methyltransferase complex of WDR5/MLL1 responsible for the methylation of H3K4me3 [5]. Moreover, the transcription of small ncRNAs is mainly transcribed by the RNA polymerase III; mutations of the core components of the enzyme complex could rescue neuronal necrosis in primary neuron culture and the fly *AG* model. These results are consistent with the increased H3K4me3 mark to induce aberrant transcription of certain small ncRNAs, including the generation of the cytotoxic tRFs. Consistent with our data, others had reported that the increase of tRF^tyr^ induced by hydrogen peroxide was dependent on the active transcription of the pre-tRNA^tyr^ by the RNA polymerase *III* [16].

In the enriched tRFs and piRNAs, we found that FAM-tRF^His-GTG^ and tRF^Gln-CTG^ showed stronger cytotoxic than the other tRFs; while, the piRNAs displayed no toxicity. Interestingly, the neurons showed swollen morphology and death, phenocopied neuronal necrosis induced by glutamate. For tRF toxicity, one study showed that the accumulation of tRF^Gly-GCC^, belonging to the subclass tiRNA-5, could decrease the cell size and increase neuronal apoptosis in mouse and human brain [54]. Our result showed that FAM-tRF^His-GTG^ and tRF^Gln-CTG^, belonging to the subclass tRF-2, displayed a direct cytotoxicity, which has not been documented before. It is likely that certain tiRNA species sensitizes neurons to stress and result in progressive neurodegeneration; whereas certain tRF-2 species are strong inducers of cell death and responsible for acute neuronal necrosis.

Our results demonstrated that nascent protein synthesis was inhibited in the early phase of neuronal necrosis induced by the cytotoxic tRFs or glutamate treatment in primary neuron cultures; this reduced protein synthesis also occurred in a mouse ischemic stroke model. In eukaryotic cells responding to environmental stress, it is common to downregulate global transcription and translation, the reduced expression of common housekeeping genes can preserve energy, while enhanced production of repair genes can promote survival. However, selective translation can be achieved by differential sensitivity of mRNAs to the changes of the translation initiation factors [55]. It has been reported that transfection of certain 5′-monophosphate-tiRNAs (such as tiRNA^val^, tiRNA^Gly^, and tiRNA^Ala^) could induce global translation suppression, formation of stress granules, and survival against mild oxidative stress in U2OS cells (human bone osteosarcoma cell line) [56]. In addition, tiRNAs generated by angiogenin cleavage could suppress apoptosis through binding with cytochrome C in response to a hyperosmotic stress in primary neurons; these tiRNAs could reduce protein synthesis [57, 58].

How inhibition of nascent protein synthesis is induced in necrosis? We propose that tRFs may reduce protein synthesis by Ago-dependent RNA silencing. It has been demonstrated that tRF^His-GTG^ and tRF^Leu-CAG^ (belong to tRF-3) could interact with the endogenous Ago2 protein and be cleaved by Ago2 in human HEK293 lysates, suggesting tRFs can generate RNAi effects similar to the miRNAs [59]. In our study, the Ago1/2 RNAi could rescue neuronal necrosis in the *AG* fly model, although this effect may be not necessarily generated from tRFs. Alternatively, tRFs have been known to directly affect the protein translation machinery. For instance, both 5′-tiRNA^Ala^ and 5′-tiRNA^Cys^ were capable to displace eIF4F from the capped mRNA by assembling unique G-quadruplex structures to suppress translation initiation [60]. Furthermore, transfection of synthetic 5′-tiRNA^Ala^ and 5′-tiRNA^Cys^ could affect the eIF2α-independent translation repression [9, 56].

Consistent with inhibition of translation, we found that the large subunit of ribosomal protein RPL26 enhanced its staining intensity in the necrotic neurons, while the small subunit of ribosomal protein RPS6 was unaltered. This phenomenon is consistent with ribosomal stalling, in which the stalled mRNA on the ribosome can be sensed by the proteins such as HBS1L, GTPBP2, and pelota (PELO) in mammalian cells. PELO recruits mammalian ATP-binding cassette protein subfamily E member 1 (ABCE1) to promote separation of the ribosomal subunits, and the released 40s small subunit can be reused; while the 60s large subunit attaches to the new nascent-chain-tRNA conjugate [29]. Ribosomal dysfunction could inhibit protein synthesis following an oxidative exposure [61, 62], and ribosome dysfunction involves in neurodegeneration [63, 64].

The important discovery in this study is that certain tRF species transcribed by RNA polymerase III, is cytotoxic (Fig. S11). Therefore, targeting the H3K4me3-mediated aberrant transcription or cytotoxic tRFs may be considered as a new strategy to suppress neuronal necrosis downstream of the NMDA receptor activation.

## Supplementary information

Supplementary figure legend

supplementary Figure 1

supplementary Figure 2-5

supplementary Figure 6-7

supplementary Figure 8-11

table S1-S47
