# Supplementary material for: Increased expression of fragmented tRNA promoted neuronal necrosis
Source: Cell Death Dis. 2021 Aug 30;12(9):823. doi: 10.1038/s41419-021-04108-6 (PMC8405691; doi:10.1038/s41419-021-04108-6)
Supplement: Supplementary file 1 — Supplementary figure legend [file 41419_2021_4108_MOESM1_ESM.docx]

**Supplementary Figure Legend**

**Figure S1.** **The length distributions of tRFs, piRNAs and miRNAs**

In primary neuron culture, Exp pep or DM pep was pretreated for 1 hour. Then, glutamate was added to induce necrosis. After 30 minutes, the total RNA was collected for sequencing analysis. y axis: total reads counts (Million); x axis: Adapter trimmed reads length (Nucleotide). The treatments are listed on the right of the chart.

**Figure S2. Transfection efficiency of small ncRNAs in the primary neuron culture**

A scramble sequence of tRF was synthesized with the addition of a FAM tag at its 5’ side (5’-FAM-tRF-random). The fluorescent labeled tRF was transfected into the embryonic rat primary neuron cultures for 30 minutes. The fluorescence was detectable in nearly 20% of cells.

**Figure S3. The type of cell death induced by tRF^Gln-CTG^ in the primary neuron culture**

After transfection of tRF^Gln-CTG^ for 12 hours, the primary neurons were stained with Annexin V and PI to label apoptotic and necrotic cell death. DAPI stains nucleus. As a control, the scrambled sequence of tRF was used. The result showed that the Annexin V signal was likely labeled the membrane debris or the broken cells, which was difficult to quantify. In contrast, the nuclei of most cells were clearly labeled by PI, indicating necrosis. The percent of PI positive cells were quantified. Five images were collected for each treatment. Trial n=2.

**Figure S4. Nascent protein synthesis quantified by high-content analysis**

In primary neuron culture, Exp pep or DM pep was pretreated for 1 hour. Then, glutamate was added to induce necrosis. After 30 minutes, the cells were fixed and assayed for nascent protein synthesis. For each trial, 25 fields were randomly selected; and the averaged fluorescent intensity was obtained automatically. Trial n=4.

**Figure S5. Characterization of tissue damage in a mouse stroke model**

(**A**) After a permanent ischemia of distal middle cerebral artery by an electric coagulation forceps for 1 hour, the cerebral blood flow was evaluated by a laser doppler flowmetry. The artery damage is on the right side, with the left side as a control. With the blood flow of the control side set as 1, the relative blood flow of the ischemic side is plotted. Trial n=4. (**B**) The same condition as **A**, the mouse brain was collected and stained with TTC. The result showed that no mitochondrial damage was detected at this time.

**Figure S6. Ribosomal protein staining in neuronal necrosis**

In primary neuron culture, Exp pep or DM pep was pretreated for 1 hour. Then, glutamate was added to induce necrosis. After 30 minutes, the cells were fixed and immune-stained for ribosomal proteins. (**A**) Example of high-content image stained with anti-RPL26 (protein component of the large subunit of ribosome), the marker of nascent protein synthesis, and DAPI. For each trial, 25 fields were randomly selected; and the averaged fluorescent intensity was obtained automatically. Trial n=3. (**B**) Example of high-content image stained with anti-RPS6 (protein component of the small subunit of ribosome), with the same condition as **A**. Trial n=1.

**Figure S7. GO and KEGG analysis of gene target of the enriched tRFs in neuronal necrosis**

To predict the mRNA targets of the enriched tRFs in neuronal necrosis, a base pairing method was applied. This method requires the predicted RNA sites to satisfy a threshold of the algorithm (miRanda and TargetScan). The GO (Gene ontology) and KEGG (Kyoto Encyclopedia of Genes and Genomes) analysis of target genes were shown.

**Figure S8. Contribution of mitochondrial fragmentation on neuronal necrosis**

In primary neuron culture, Mdivi-1 was pretreated for 40 minutes. Then, glutamate was added to induce necrosis. After 4 hours, the cells were collected for the LDH assay. Trial n=4.

**Figure S9. Confirmation of *Ago* RNAi effect on flies**

The *Ago1*RNAi and *Ago2* RNAi lines were crossed with the *Actin-Gal4*, a pan-cell promoter, respectively. qRT-PCR was performed to determine the knockdown effect of the RNAi. With the *Actin-Gal4* crossed with “TB00073” (the background fly to generate RNAi lines) as the control, the relative mRNA level of Ago1 and Ago2 were shown for the *Actin*>*Ago1RNAi* and *Actin*>*Ago2RNAi*. Trial n=3.

**Figure S10. qRT-PCR to determine the GluR1^Lc^ expression in the fly lines**

The following fly lines were tested for the GluR1^Lc^ expression, including control (the progeny flies of the AG crossed with TB00072, genotype: *Appl-Gal4/+; UAS-GluR1^Lc^, tubulin-Gal80^ts^*/+; +/+), RanRNAi (genotype: *Appl-Gal4*/+; *UAS-GluR1^Lc^*, *tubulin-Gal80^ts^*/*+*; *UAS-RanRNAi* /+), *Ago1RNAi* (genotype: *Appl-Gal4*/+; *UAS-GluR1^Lc^*, *tubulin-Gal80^ts^*/*UAS-Ago1RNAi*; +/+), *Ago2RNAi* (*Appl-Gal4*/+; *UAS-GluR1^Lc^*, *tubulin-Gal80^ts^*/*UAS-Ago2RNAi*; +/+), *w^1118^* (the progeny flies of the AG crossed with *w^1118^*, genotype: *Appl-Gal4/+; UAS-GluR1^Lc^, tubulin-Gal80^ts^*/+; +/+), *Ran^G19V,Q69L^*(genotype: *Appl-Gal4*/+; *UAS-GluR1^Lc^*, *tubulin-Gal80^ts^*/*Ran^G19V,Q69L^*; +/+). These lines are all under the AG background. Without heat shock (HS), the GluR1^Lc^ transgene should not be expressed. With HS for 24 hours at 30 °C, the GluR1^Lc^ transgene were expressed in all of the lines. Unpaired *t*-test, ** for *P*<0.01; *** for *P*<0.001.

**Figure S11. Our proposed model of neuronal necrosis**

Upon calcium overload, the increase of H3K4me3 promotes the expression of tRFs, which are transported by the RanGTPase from nucleus to cytosol. Then, the tRFs bind with the Ago1/2 proteins to induce ribosomal stalling. The translation of proteins function in mitochondrial metabolism is likely preferentially downregulated, which results in mitochondrial fragmentation and neuronal necrosis.
