# Supplementary figures and images for: Increased expression of fragmented tRNA promoted neuronal necrosis
Source: Cell Death Dis. 2021 Aug 30;12(9):823. doi: 10.1038/s41419-021-04108-6 (PMC8405691; doi:10.1038/s41419-021-04108-6)

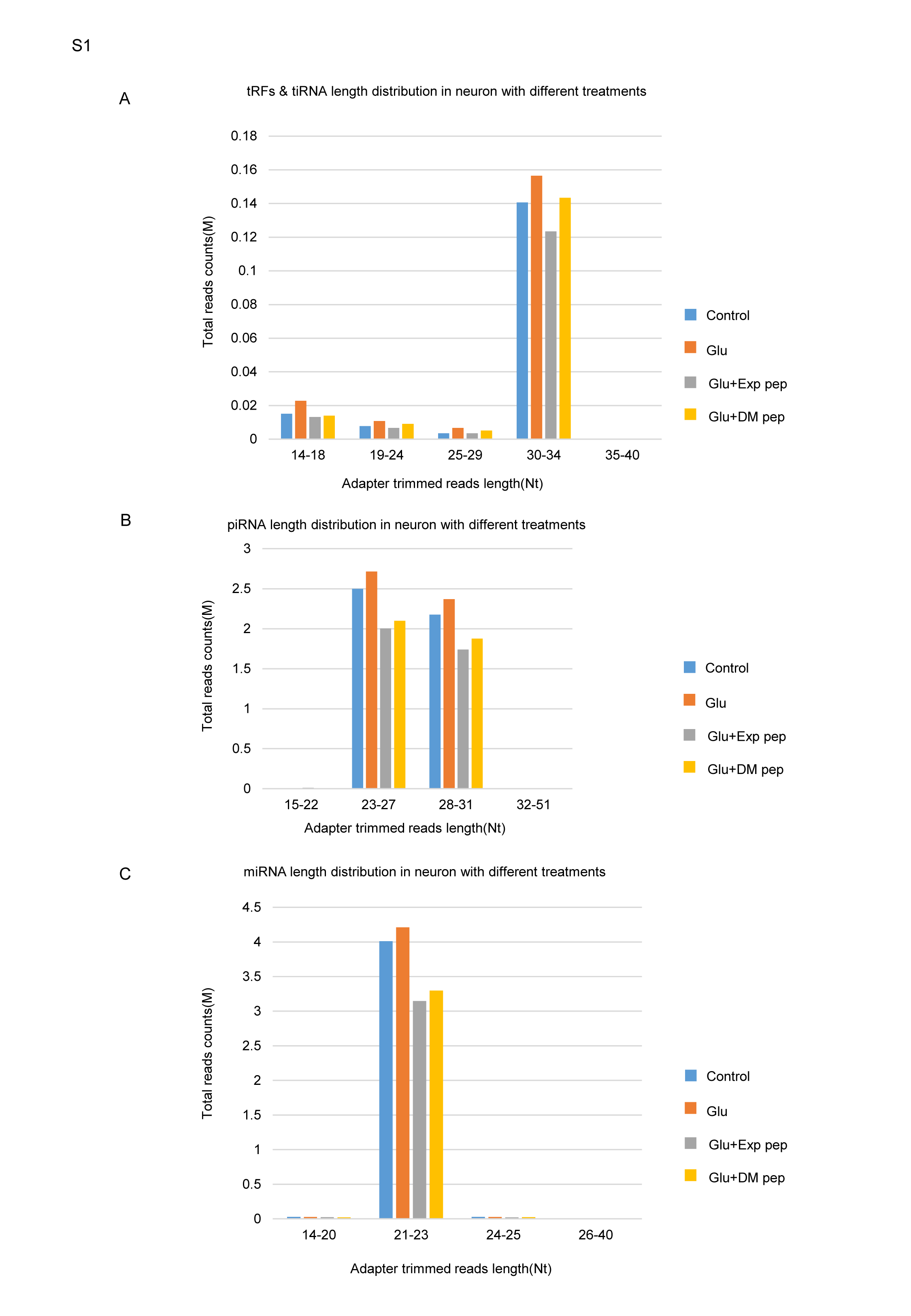

Supplement: Supplementary file 2 — supplementary Figure 1 [file 41419_2021_4108_MOESM2_ESM.tif]

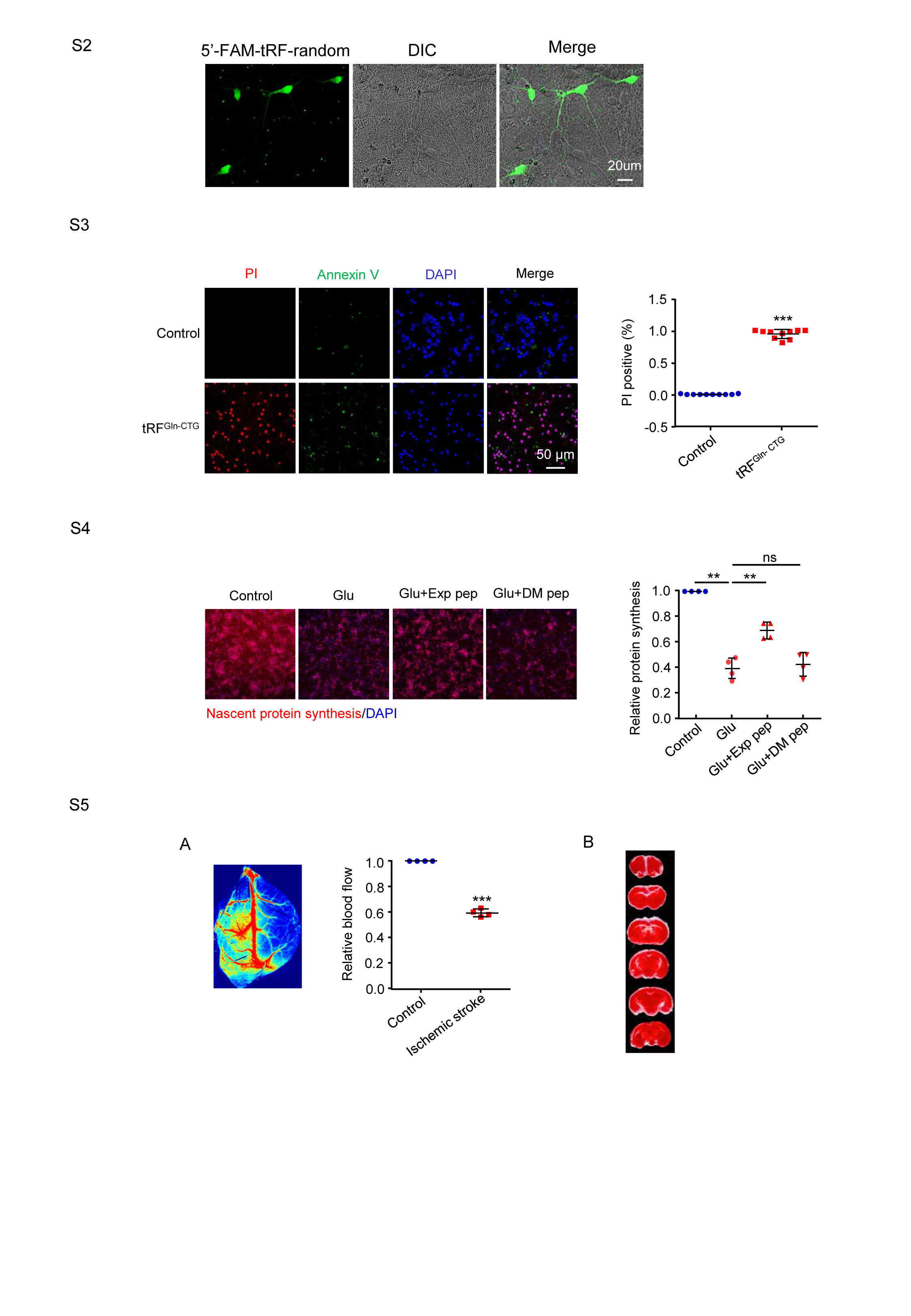

Supplement: Supplementary file 3 — supplementary Figure 2-5 [file 41419_2021_4108_MOESM3_ESM.tif]

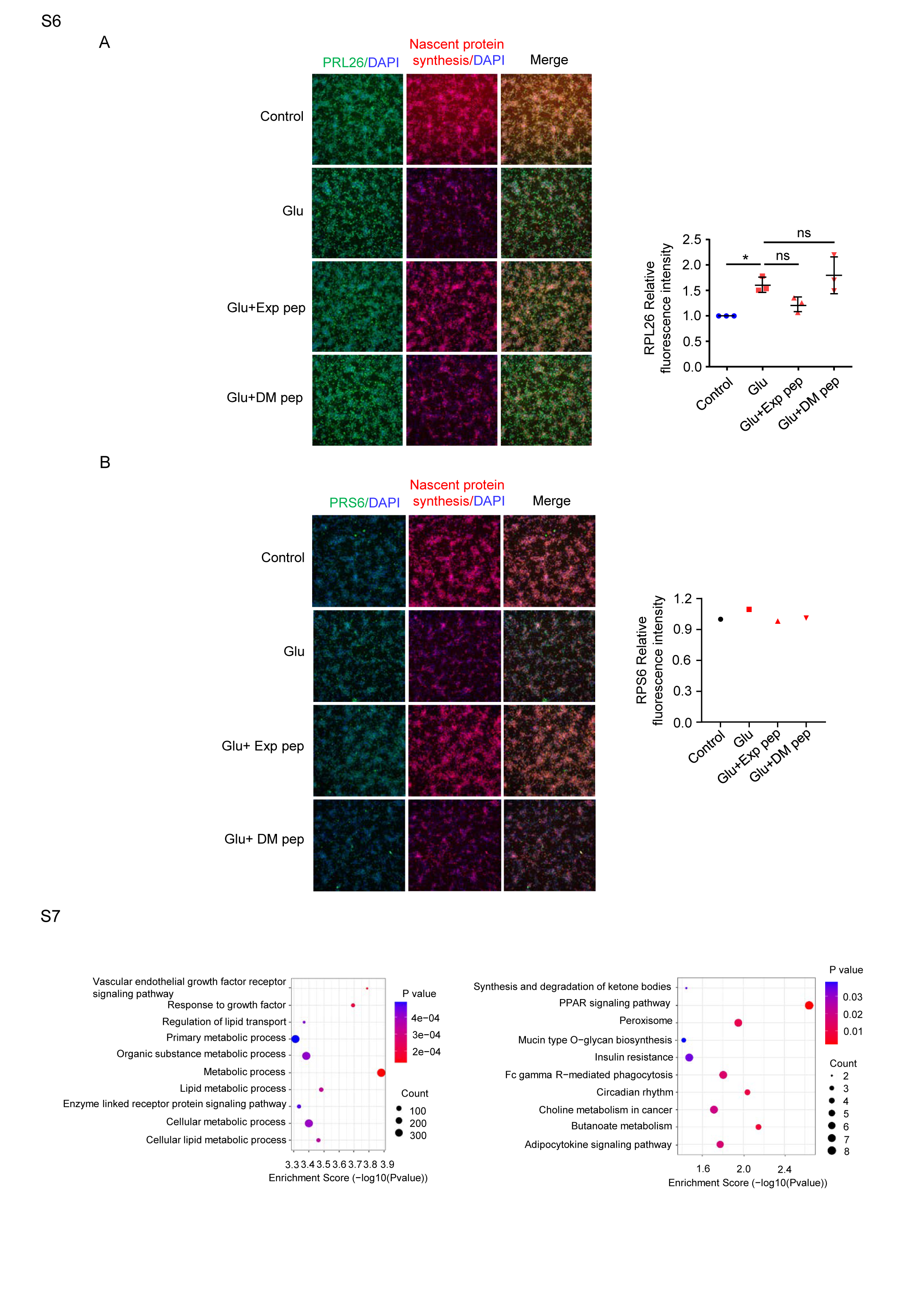

Supplement: Supplementary file 4 — supplementary Figure 6-7 [file 41419_2021_4108_MOESM4_ESM.tif]

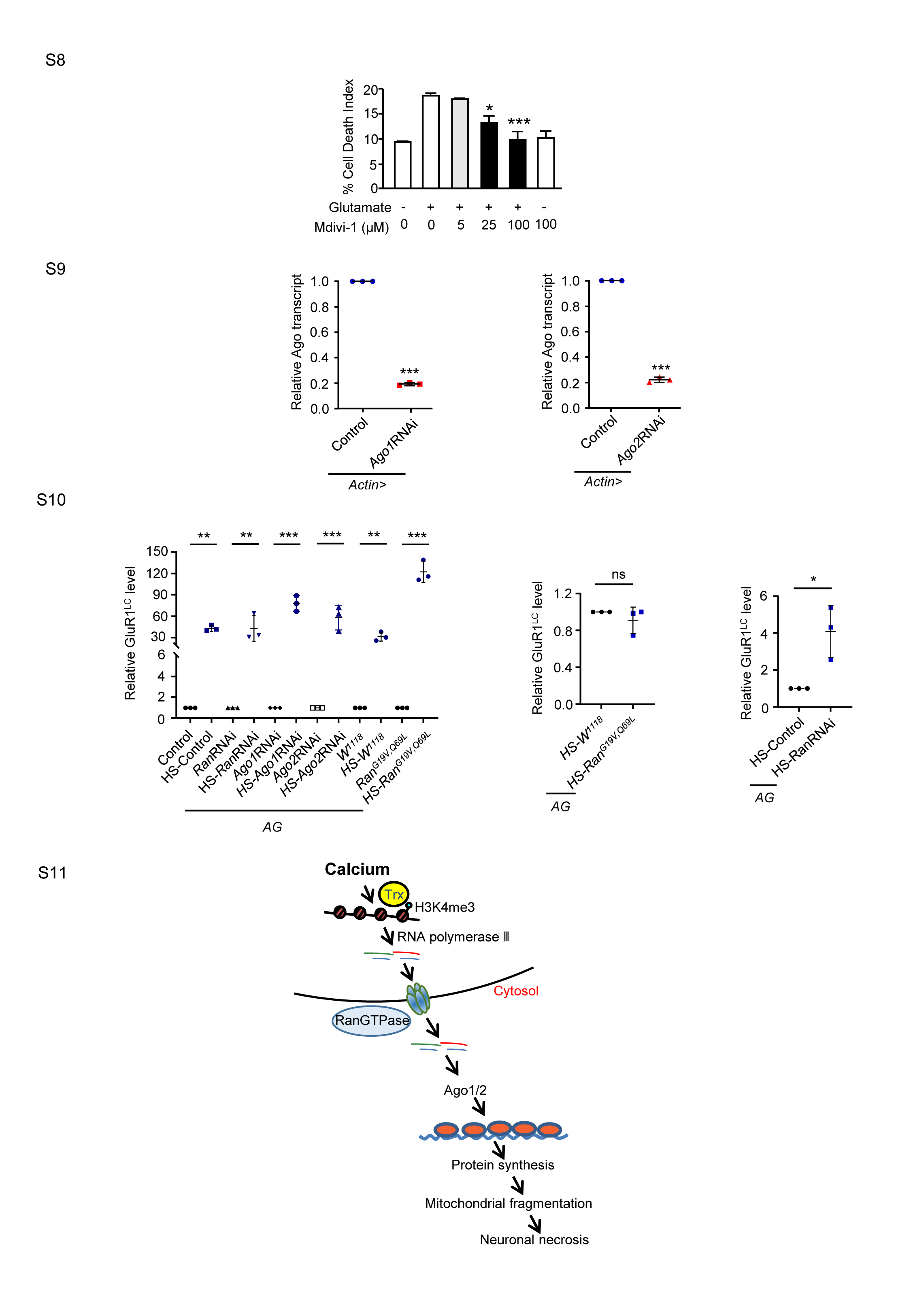

Supplement: Supplementary file 5 — supplementary Figure 8-11 [file 41419_2021_4108_MOESM5_ESM.tif]
